# Formulating Bioactive Terpenes

**DOI:** 10.3390/biom11121745

**Published:** 2021-11-23

**Authors:** Ádley A. N. Lima, Letícia S. Koester, Valdir F. Veiga-Junior

**Affiliations:** 1 Programa de Pós-Graduação em Ciências Farmacêuticas, Departamento de Farmácia, Universidade Federal do Rio Grande do Norte, Av. General Cordeiro de Farias, s/n, Petrópolis, Natal 59012-570, Brazil; 2 Programa de Pós-Graduação em Ciências Farmacêuticas, Faculdade de Farmácia, Universidade Federal do Rio Grande do Sul, Av. Ipiranga, 2752, Porto Alegre 90610-000, Brazil; 3 Departamento de Engenharia Química, Instituto Militar de Engenharia, Praça Gen. Tibúrcio, 80, Praia Vermelha, Urca, Rio de Janeiro 22290-270, Brazil

Terpenes are specialized metabolites mainly produced by plants and are highly bioactive. Incense and myrrh are examples of terpene-rich oleoresin materials among the older natural products used by humankind. They were already present in Hippocrates’ ancient Greek medicines and also as two of the three holy gifts from the three wise men at Christmas celebrating the birth of Christ. Natural oleoresins and their volatile monoterpenes and sesquiterpenes, together with the resinous diterpenes and triterpenes, are still used for therapeutic purposes in traditional populations, being burned to alleviate migraine, as an anti-inflammatory, for healing and for several other medicinal uses. Indeed, modern natural products’ chemistry, pharmacology and formulation processes are a vast area. Amyrin triterpenes from Burseraceae breu and caryophyllene sesquiterpenes from copaiba oils (*Copaifera* sp.) are resources from Amazonian biodiversity still yet to be fully developed [1,2,3,4].

The importance of these studies to one of the most important terpenes, the diterpene docetaxel, can be observed. For several decades, the chemistry of producing this potent anti-tumoral agent was developed together with all clinical studies to to faster achieve the market. It has been saving thousands of people, but its low solubility has demanded formulation with tween or castor oil, resulting in hemolysis with consequent hypersensitivity reactions. The development of liposomal and other drug delivery systems was very successful in reducing the toxicity of several drugs, including terpenes such as docetaxel [5,6,7].

Terpenes are also used as sweeteners and perfumes; as green industrial solvents; as modern cosmetics that act in cannabinoid receptors; and, also, as new drugs. The scope of this Special Issue of *Biomolecules*, “Evaluation and formulation of Bioactive Terpenes”, was to provide a current view on natural products’ chemistry and pharmacology, and especially on modern drug delivery systems of monoterpenes, sesquiterpenes, diterpenes and triterpenes. All these main terpenic classes were included in seven papers published.

The review article specially written for this Special Issue titled “Essential Oils and Isolated Terpenes in Nanosystems Designed for Topical Administration: A Review” [8] presented a panorama of growing research that corroborates the benefits of encapsulating either terpenes or the essential oils containing them in different nanostructure systems: polymeric, lipidic or molecular complexes. In most studies (66%), terpenes are the bioactive compounds to which encapsulation brings advantages such as chemical protection and improved bioefficacy. This is exemplified by β-caryophyllene, a sesquiterpene present in high amounts in Copaifera multijuga Hayne, which has been extensively investigated by our research group. Nanoemulsification of C. multijuga oil showed a protective effect on β-caryophyllene under stressing conditions [9], promoted its penetration through skin (in vitro model) [10] and potentiated the anti-inflammatory activity [4,11]. The review article also sheds light on the use of terpenes associated with nanosystems as permeation promoters that make up 34 % of the studies. The association of terpenes, ethanol and phospholipids that form the penetration-enhancing elastic and deformable “invasomes” stands out. Interestingly, monoterpenes rather than larger terpenes are usually chosen to play the role of the “excipient” (Figure 1) [8].

The monoterpene class was also addressed by Danielli et al. [12] in a paper published in this Special Issue. The authors evaluated the influence of seasonal variations on the chemical composition of essential oils extracted from *Nectandra megapotamica* (Spreng.) Mez (Lauraceae). Samples extracted in the pre-blooming and blooming phases presented higher percentages of monoterpenes, which correlated with higher antifungal and lower antichemotactic effects, revealing the importance of this class to antifungal activity, usually attributed to the presence of oxygenated sesquiterpenes.

A study published in this Issue by da Silva et al. [13] showed the in vitro activity of the triterpenes betulinic acid and ursolic acid and twenty of their derivatives against planktonic and biofilm cells (Gram-positive bacterial pathogens: *Enterococcus faecalis*, *Staphylococcus aureus* and *S. epidermidis*). The authors verified, for the first time, whether different groups of triterpenes at carbon 3 (C-3) may interfere in the antibiofilm activity with minimal or no antibacterial effect. After the screening of 22 compounds at 3 distinct concentrations, they found antibiofilm activity for 8 distinct derivatives without an antibiotic effect.

Another study with important triterpenes was published in this Issue by da Silva Júnior et al. [14], developing inclusion complexes of α, β amyrin, a natural mixture of pentacyclic triterpenes with cyclodextrin, by two different methods. Physicochemical characterization indicated the formation of inclusion complexes with cyclodextrin which were shown to be an effective and promising alternative to enhance the anti-inflammatory activity and safety of this triterpene.

Bioactive diterpenes were also described in this Special Issue: a review and an anti-tumor study with cembranoids from tobacco [15,16], and glucoside derivative rebaudioside C, a sweetener [17].

The cembranoids present an unusual 14-member macrocyclic diterpenic backbone, with three symmetrically distributed methyl groups and one isopropyl; they usually present a symmetry plane. Very common in tobacco leaves, but also found in *Pinus* and soft corals, they present several biological activities, highlighting the neuroprotective action blocking the functions of nicotinic acetylcholine receptors, with singular cellular signal transduction pathways and anti-tumoral properties. These activities were reviewed in one paper from this Special Issue [15], while another paper [16] described the isolation of cembranoids from tobacco leaves and the evaluation against hepatoma cell lines, observing that some of them can alter cell morphology and membrane permeability before inducing apoptosis.

This wide biological property profile that turns terpenes into one of the most important classes of natural products is demonstrated in another paper published in this Special Issue. “Enzymatic Synthesis and Characterization of a Novel α-1→6-Glucosyl Rebaudioside C Derivative Sweetener” [17] brings forward this unusual sweetener characteristic from diterpenes, which are so important in food sciences in times of epidemic metabolic syndrome. In this paper, the authors present some enzymatic transformations on rebaudioside C, the third most prevalent steviol glycoside in *Stevia rebaudiana* leaves, to increase its applications in food and beverage products with improved sweetness, reduced bitterness and enhanced solubility in water.

## Figures and Tables

**Figure 1:**
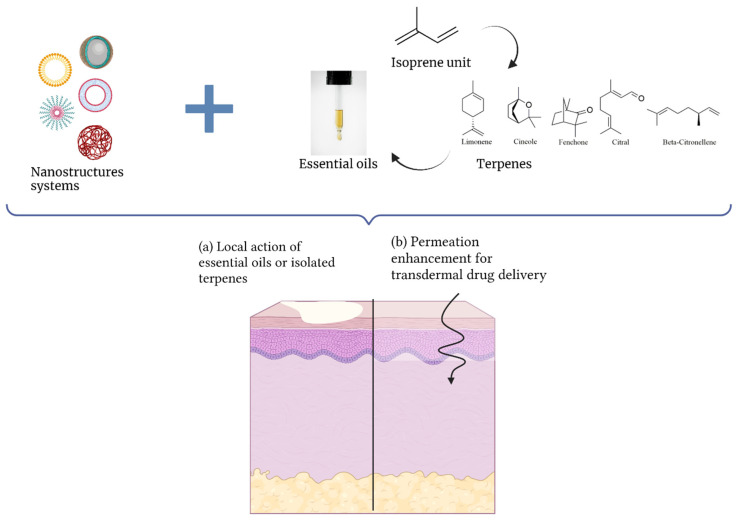
Association of terpenes or essential oils containing them in nanostructured systems with two purposes upon topical application: (**a**) as a bioactive and (**b**) as a permeation enhancer.
